# Expression and Secretion of TNF-α in Mouse Taste Buds: A Novel Function of a Specific Subset of Type II Taste Cells

**DOI:** 10.1371/journal.pone.0043140

**Published:** 2012-08-14

**Authors:** Pu Feng, Hang Zhao, Jinghua Chai, Liquan Huang, Hong Wang

**Affiliations:** 1 Monell Chemical Senses Center, Philadelphia, Pennsylvania, United States of America; 2 Pulmonary and Critical Care Unit, Department of Medicine, Massachusetts General Hospital and Harvard Medical School, Boston, Massachusetts, United States of America; German Institute of Human Nutrition Potsdam-Rehbruecke, Germany

## Abstract

Taste buds are chemosensory structures widely distributed on the surface of the oral cavity and larynx. Taste cells, exposed to the oral environment, face great challenges in defense against potential pathogens. While immune cells, such as T-cells and macrophages, are rarely found in taste buds, high levels of expression of some immune-response-associated molecules are observed in taste buds. Yet, the cellular origins of these immune molecules such as cytokines in taste buds remain to be determined. Here, we show that a specific subset of taste cells selectively expresses high levels of the inflammatory cytokine tumor necrosis factor-α (TNF-α). Based on immuno-colocalization experiments using taste-cell-type markers, the TNF-α-producing cells are predominantly type II taste cells expressing the taste receptor T1R3. These cells can rapidly increase TNF-α production and secretion upon inflammatory challenges, both *in vivo* and *in vitro.* The lipopolysaccharide (LPS)-induced TNF-α expression in taste cells was completely eliminated in *TLR2^−/−^/TLR4^−/−^* double-gene-knockout mice, which confirms that the induction of TNF-α in taste buds by LPS is mediated through TLR signaling pathways. The taste-cell-produced TNF-α may contribute to local immune surveillance, as well as regulate taste sensation under normal and pathological conditions.

## Introduction

Taste dysfunction impacts negatively on quality of life and general wellbeing of patients. While the mechanism of taste impairments associated with various pathological conditions remains largely unclear, clinical observations and research using experimental models suggest that inflammation may contribute to the development of taste disorders. For example, patients with chronic inflammatory and autoimmune diseases may develop taste dysfunction [1], [2]. The exogenous use of cytokines, such as interferons (IFNs), can cause taste abnormalities in patients [3]. In experimental models, IFNs induce apoptosis of taste cells [4]. Inflammatory activators, such as lipopolysaccharide (LPS) from bacteria, can affect taste progenitor cell proliferation, taste cell turnover, and recovery of taste nerve responses after nerve section and dietary sodium restriction [5], [6]. It is also observed that an increase of IL-1β in the tongue following injury of the chorda tympani nerve has a beneficial effect on taste function [7]. These studies indicate an interaction between the gustatory system and immune responses under various conditions.

Recent studies have also revealed some interesting yet not well-documented immunologic features of taste buds. Taste buds are located in numerous taste papillae on the surface of the tongue, as well as in the epithelium of the soft palate and larynx. Very few, if any, leukocytes are found in healthy taste buds, although an array of immune cells regularly reside in the epithelium and lamina propria surrounding taste buds [8], [9]. On the other hand, taste buds appear to be self-equipped with various immune mechanisms, especially those in the innate arm of the immune system. For example, many components of immune or inflammatory signaling pathways are highly expressed in taste buds, including cytokines and their receptors, chemokines and their receptors, components of the complement system, and Toll-like receptors (TLRs) [4], [5], [10], [11]. These studies raise the possibility that taste cells may play a role in oral mucosal immunity.

Several cytokines, including tumor necrosis factor-α (TNF-α), have important roles in modulating various physiological processes, as well as in mediating immune responses and inflammation [12]. TNF-α and its receptors are known to regulate a variety of cellular signaling pathways that affect cell growth, proliferation, differentiation, and survival [13]. TNF-α was thought to be produced primarily by macrophages, but it is also produced by a broad variety of cell types, including lymphoid cells, mast cells, endothelial cells, cardiac myocytes, adipocytes, fibroblasts, and neurons [14]–[16]. In the oral cavity, TNF-α has been found in salivary glands and saliva [17]–[19]. Although TNF-α expression was observed in taste buds [5], it is unclear what specific type of cells produces TNF-α and whether TNF-α is released from taste cells to regulate neighboring tissues including oral mucosa.

In this study, we investigated the expression, production, and release of TNF-α in taste buds and compared them with those in nontaste oral epithelium. Our results show that taste buds in all three types of tongue taste papillae highly express TNF-α in untreated control mice and identify a subset of type II taste cells as the major TNF-α-producing cells. In response to inflammatory challenges, taste bud cells can greatly increase the production and secretion of TNF-α, both *in vivo* and *in vitro*. The lipopolysaccharide (LPS)-induced TNF-α expression in taste cells was completely eliminated in *TLR2^−/−^/TLR4^−/−^* double-gene-knockout mice, which confirms that the induction of TNF-α production in taste buds by LPS is mediated through TLR signaling pathways. These findings revealed a potential immune-regulatory function of a subset of taste cells.

## Results

### Mouse Taste Buds Highly Express TNF-α

**Figure 1:**
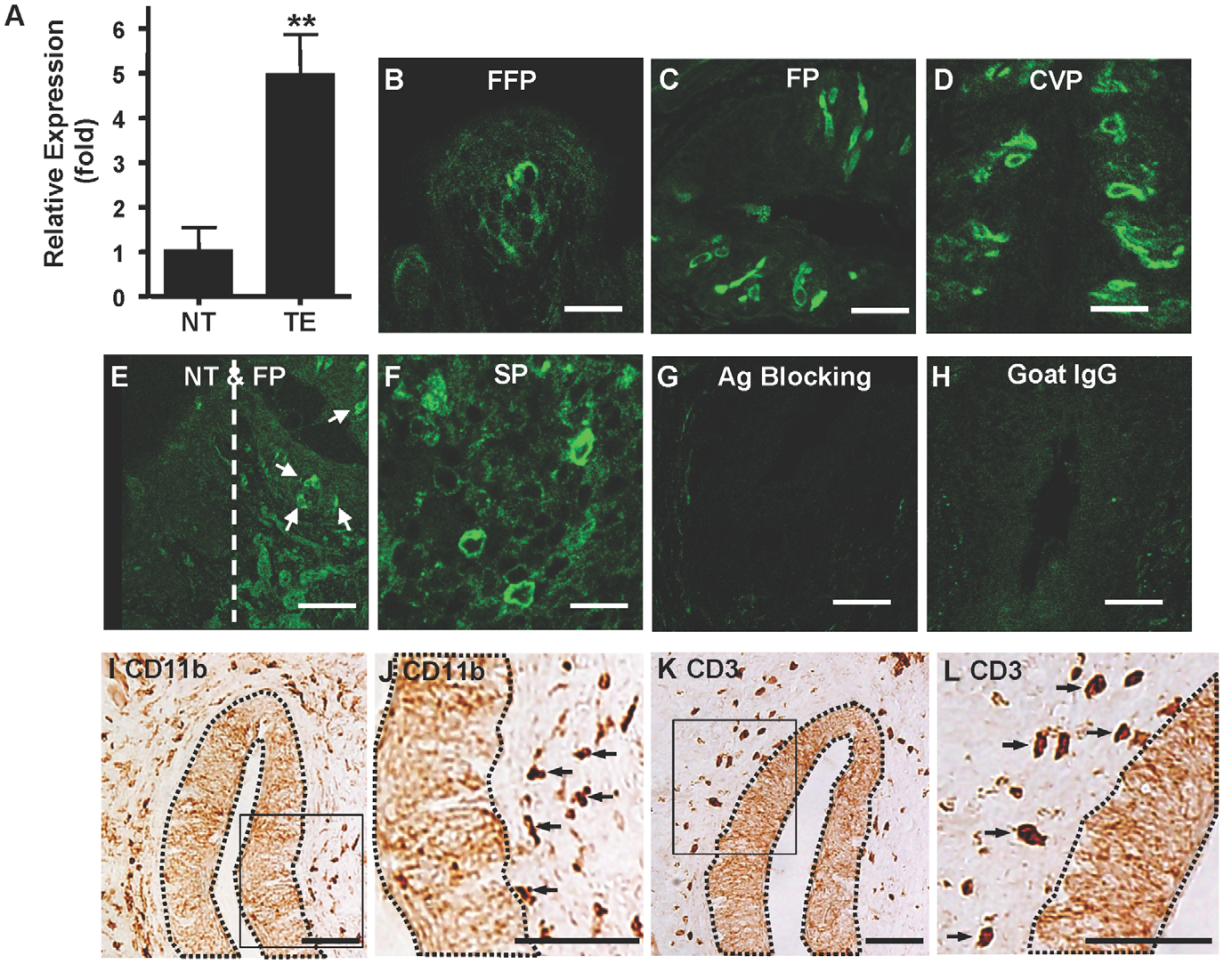
Expression of TNF-α in the taste epithelium of control adult mice. (**A**) qRT-PCR analysis of TNF-α expression in taste (TE) and nontaste (NT) epithelium: relative expression levels (fold) of the *TNF-α* gene. β-Actin served as the endogenous control gene for relative quantification. Epithelial tissues from 3 mice were pooled for each set of RNA sample preparation. Four independent sets of samples were established, and totally 12 mice were used in this study. Data are mean ±SD. **P<0.01 (t = 7.8, df = 6). (**B–D**) Confocal images of specific immunofluorescent staining of TNF-α in taste buds of fungiform papillae (FFP) (**B**), foliate papillae (FP) (**C**), and circumvallate papillae (CVP) (**D**). (**E**) No TNF-α expression was observed in nontaste epithelium (NT) adjacent to foliate papillae (FP). Arrows indicate TNF-α-positive cells in foliate taste buds. (**F**) Spleen (SP) was used as a positive-tissue control. (**G**) The specificity of the TNF-α antibody was determined by blocking the antibody with recombinant TNF-α protein before staining (Ag Blocking). (**H**) The specificity of the secondary antibody was tested by immunostaining with nonspecific goat IgG instead of the TNF-α antibody (Goat IgG). (**I–L**) Antibodies against CD11b (**I, J**) and CD3 (**K, L**) were used to identify macrophages and granulocytes (CD11b), as well as T lymphocytes (CD3), on circumvallate sections. Brown solid staining in connective tissues indicates specifically stained cells. Dotted lines indicate areas of taste epithelium in circumvallate papillae. Boxed regions in **I** and **K** are shown in **J** and **L**, respectively, as enlarged images. These images show that CD11b- and CD3-positive cells are in connective tissue layers but not in taste buds. Arrows indicate representative CD11b- and CD3-positive cells. Ten mice were used for panels **B**–**F** and **I**–**L**, and five were used for panels **G–H**. Scale bars: 10 µm (**B**), 25 µm (**C, D, F**), and 50 µm (**E, G–L**).

To investigate the expression of TNF-α in taste tissues, we first analyzed the level of *TNF-α* mRNA in mouse circumvallate and foliate epithelium by quantitative reverse-transcription polymerase chain reaction (qRT-PCR) and compared this level with that in lingual epithelium devoid of taste buds. As shown in Figure 1A, the *TNF-α* mRNA level in the taste epithelium was about 5-fold higher than that in nontaste lingual epithelium of mice. The results demonstrate that the peripheral gustatory epithelium has high potential for TNF-α production even in control mice that appeared healthy.

The production and presence of TNF-α in taste tissues were further confirmed by immunohistochemistry using a specific antibody against TNF-α. We examined frozen tongue sections containing fungiform, foliate, or circumvallate papillae. As shown in Figure 1B–D, TNF-α immunoreactivity was evident in taste buds of all three papilla types, with TNF-α-immunoreactive cells displaying typical morphology of taste bud cells. In contrast, no TNF-α immunoreactivity was observed in epithelium of nontaste tissue immediately adjacent to taste buds (Figure 1E). As a positive control, we stained mouse spleen sections with the TNF-α antibody (Figure 1F). Specificity of the antibody was shown by preincubation of TNF-α antibody with recombinant mouse TNF-α protein, which eliminated staining of taste bud cells (Figure 1G). Species- and isotype-matched nonspecific goat immunoglobulin G (IgG) paired with the same secondary antibody as the TNF-α immunostaining showed no signals above background level in taste buds (Figure 1H). To exclude the possibility that the TNF-α-immunoreactive cells in taste buds were infiltrating TNF-α-producing leukocytes, we used specific antibodies against CD11b (a marker for macrophages and granulocytes such as activated neutrophils) and CD3 (a marker for T-lymphocytes) on tongue sections and found no evidence of infiltration of these cells into taste buds (Figure 1I–L). The results demonstrate that the taste bud in mouse fungiform, foliate, and circumvallate papillae is a highly specialized structure in the oral cavity that can produce TNF-α in addition to its basic functions in taste signaling. The cumulative evidence indicates that taste bud TNF-α indeed originates from taste cells themselves rather than from infiltrating leukocytes.

### A Subset of Type II Taste Cells Produces TNF-α

**Figure 2:**
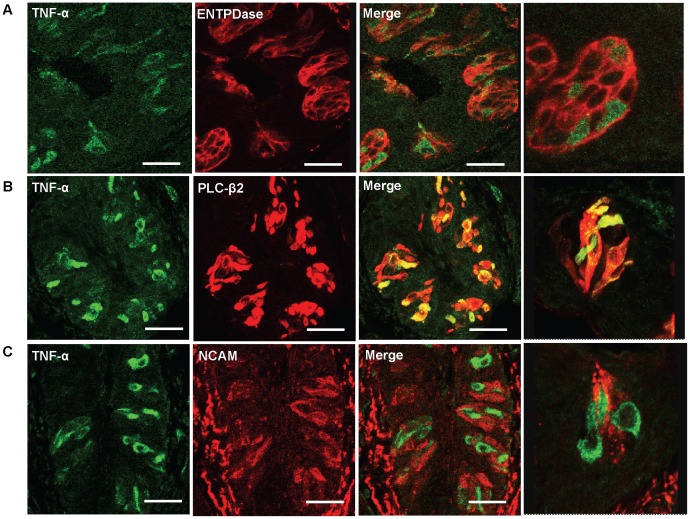
Identification of TNF-α-producing cells in taste buds. Confocal images of double-immunofluorescent staining of TNF-α (green) and different taste cell type markers (red) on foliate papillae sections of control C57BL/6 mice, with high-magnification images shown in the far right panels. (**A**) Double immunostaining of TNF-α (green) and type I taste cell marker ectonucleotidase nucleoside triphosphate diphosphohydrolase (ENTPDase; red), showing no TNF-α expression in type I taste cells. (**B**) Double immunostaining of TNF-α (green) and type II taste cell marker phospholipase C-β2 (PLC-β2; red), showing TNF-α expression in a subset of type II taste cells. Note that all the TNF-α-producing cells express PLC-β2, and about half of PLC-β2-positive cells co-express TNF-α. (**C**) Double immunostaining of TNF-α (green) and type III taste cell marker neural cell adhesion molecule (NCAM; red), showing no TNF-α expression in type III taste cells. Five mice were included in each group of the experiment. Scale bars: 20 µm (**A**) and 30 µm (**B** and **C**).

Taste buds contain at least three types of mature taste cells: type I cells that may function as supporting cells; type II cells that are responsible for sweet, bitter, and umami taste perception; and type III cells that form conventional synapses with gustatory nerve fibers [20]. Each of these three classes of taste cells can be identified by different biomarkers that are expressed specifically by these cells. To study what types of taste cells produce TNF-α, we performed immuno-colocalization experiments using antibodies against TNF-α and various taste-cell-type markers. As shown in Figure 2, TNF-α was exclusively expressed in a subset of type II taste cells labeled by phospholipase C-β2 (PLC-β2) and was not detected in type I and type III cells labeled by ectonucleotidase nucleoside triphosphate diphosphohydrolase 2 (ENTPDase2) and neural cell adhesion molecule (NCAM), respectively. TNF-α immunoreactivity was found only in type II taste cells in all three types of taste papillae: circumvallate (Figure 2), fungiform, and foliate (data not shown). The results indicate that type II taste cells are the exclusive cellular source for TNF-α produced in taste buds. It is also noteworthy that almost all TNF-α-producing cells in taste buds are PLC-β2 positive, whereas 46.8% and 37.3% of PLC-β2-positive cells in taste buds of foliate and circumvallate papillae express TNF-α, respectively. These observations suggest that PLC-β2-expressing type II taste cells are heterogeneous and can be divided into two subsets based on TNF-α expression: PLC-β2^+^TNF-α^+^ and PLC-β2^+^TNF-α^-^. We therefore further characterized the TNF-α-producing type II cells in the taste epithelium.

### TNF-α is Selectively Produced by T1R3-expressing Type II Taste Cells

**Figure 3:**
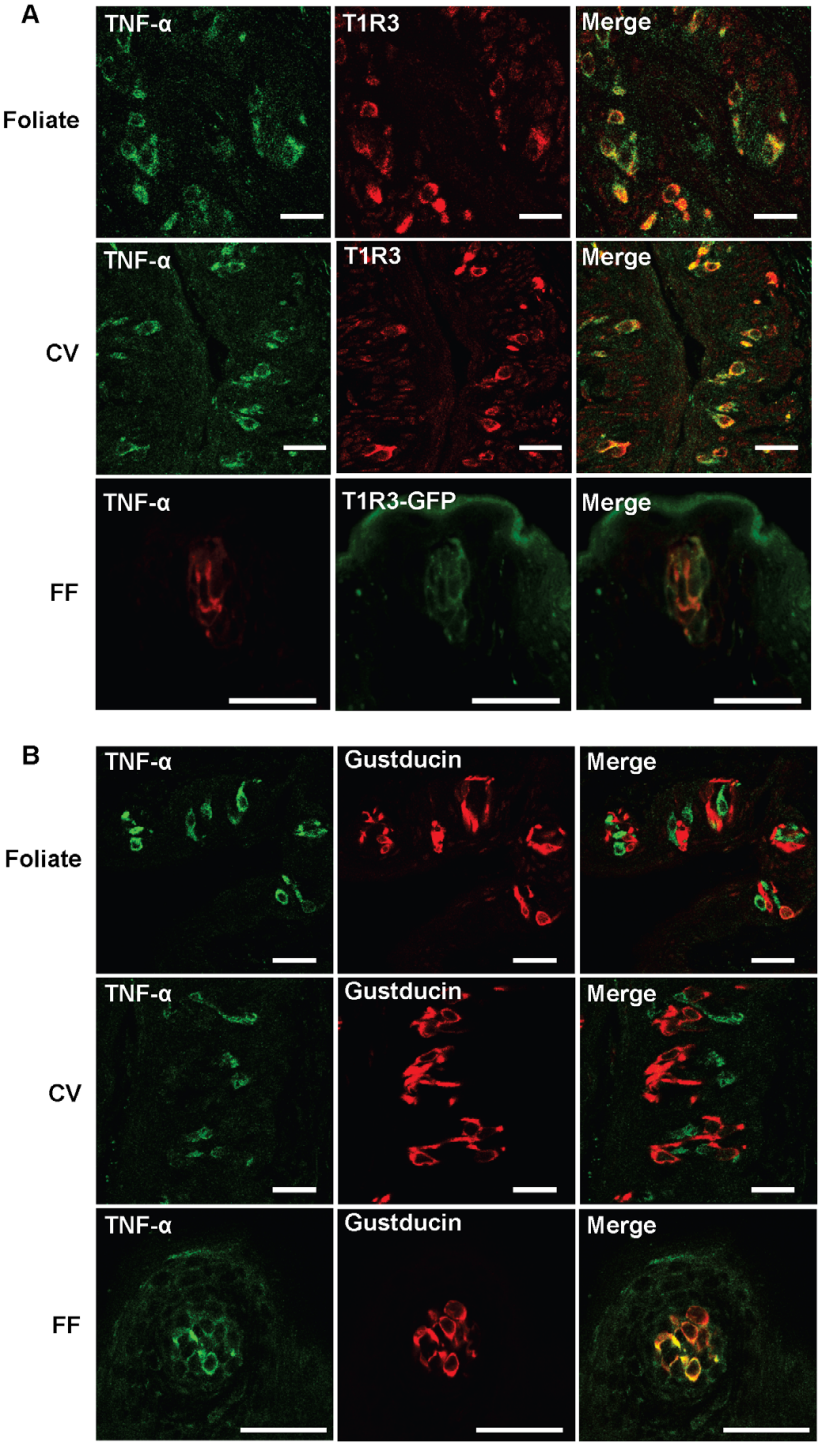
Identification of specific type II taste cells that express TNF-α in mouse taste buds. Confocal images of immunofluorescent staining of TNF-α and markers for different subtypes of type II taste cells on taste papillae sections of control C57BL/6 mice. (**A**) Top and middle panels: double immunostaining for TNF-α (green) and T1R3 (red), showing TNF-α expression in T1R3-positive taste cells in foliate (top) and circumvallate (CV; middle) papillae. Bottom panels: TNF-α immunostaining (red) in a fungiform (FF) papilla from a T1R3-GFP (green fluorescent protein) mouse. (**B**) Double immunostaining of TNF-α (green) and gustducin (red), showing the difference in TNF-α expression in gustducin-positive type II taste cells in foliate, circumvallate (CV), and fungiform (FF) papillae. Five mice were included in each group of the experiment. Scale bars: 35 µm.

Expression of G-protein-coupled T1R and T2R taste receptors and their downstream signaling molecules such as gustducin (a G-protein α subunit) is one of the most important features of type II cells, because these components are not present in type I or type III taste cells. However, type II cells are heterogeneous in function and in expression of taste receptors. Umami, sweet, and bitter cells express umami (T1R1/T1R3), sweet (T1R2/T1R3), and bitter (T2Rs) receptor molecules, respectively. The expression of G-protein subunits, such as gustducin, is also heterogeneous among type II cells. Gustducin and T1R3, a critical subunit of umami and sweet taste receptors, are co-expressed in fungiform but are mostly segregated in foliate and circumvallate taste buds (Figure S1) [21], [22]. To further investigate the subpopulation of type II taste cells that produce TNF-α, we analyzed the co-expression of TNF-α with T1R3 and gustducin. As shown in Figure 3A, TNF-α and T1R3 expression is almost completely colocalized in taste cells of foliate, circumvallate, and fungiform papillae. Same results were obtained using a T1R3 specific antibody and T1R3-GFP (expressing green fluorescent protein under the control of a mouse T1R3 promoter) transgenic mice (data not shown for foliate and circumvallate papillae in T1R3-GFP mice). In contrast, TNF-α is not expressed in gustducin-positive cells in foliate and circumvallate taste buds but is colocalized with gustducin in fungiform taste buds (Figure 3B), in strong agreement with the co-expression patterns of T1R3 and gustducin in the three types of taste papillae as shown in Figure S1 and as published in previous studies [21], [22]. These data strongly suggest that T1R3-expressing type II taste receptor cells are the cellular source of TNF-α in taste buds.

### Systemic Inflammation Induces TNF-α Expression in Taste Buds

**Figure 4:**
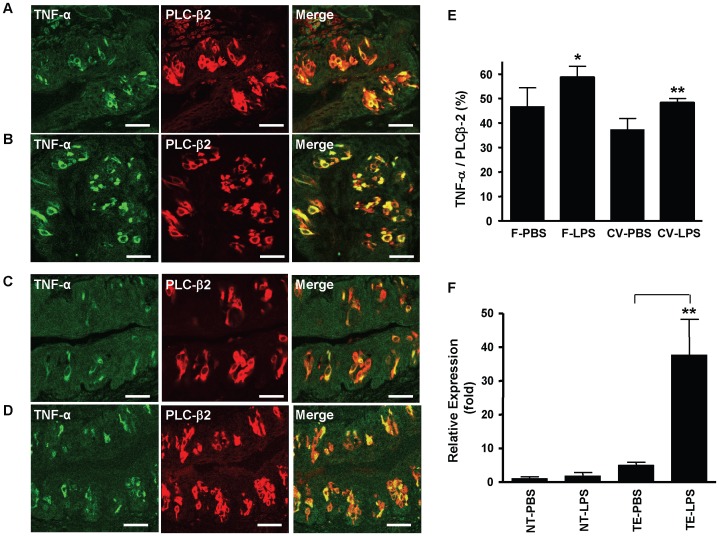
Systemic administration of microbial LPS increases TNF-α expression in taste cells. (**A–D**) Confocal images of double-immunofluorescent staining of TNF-α (green) and the type II taste cell marker PLC-β2 (red) on tissue sections of foliate (**A, B**) and circumvallate (**C, D**) papillae collected 3 h after PBS (vehicle control; **A**, **C**) or LPS (5 mg kg^−1^ body weight, i.p.; **B**, **D**) injection. Scale bars: 35 µm. (**E**) Numbers of TNF-α-positive cells and PLC-β2-positive cells from foliate (F) and circumvallate (CV) sections obtained from PBS- or LPS-treated mice. The percentage of TNF-α-positive cells in the population of PLC-β2-positive cells was calculated and plotted (TNF-α/PLC-β2 (%)). Data are mean ±SD (n = 5 mice). *P<0.05 (t = 3.2, df = 8), **P<0.01 (t = 5.3, df = 8), compared with PBS groups. (**F**) qRT-PCR analysis of TNF-α expression in taste epithelium (TE) containing circumvallate and foliate taste buds and in nontaste lingual epithelium (NT) of PBS- and LPS-treated mice. Relative expression levels (fold) of the *TNF-α* gene are shown. *TNF-α* expression level in nontaste epithelium of control mice (NT-PBS) was set to 1. β-Actin served as the endogenous control gene for relative quantification. For each mouse group, epithelial tissues from 2–3 mice were pooled for each set of RNA sample preparation. Four independent sets of samples were prepared, and totally 18 C57BL/6 mice were used in this study. Data are mean ±SD. **P<0.001 (t = 6.1, df = 6), compared with the PBS group.

After determining the identity of TNF-α-producing taste cells, we further asked whether pro-inflammatory agents can induce TNF-α production in taste buds in vivo. For this purpose, we injected C57BL/6 mice intraperitoneally (i.p.) with LPS (5 mg kg^−1^ body weight) [4], a gram-negative bacterial cell-wall component that induces systemic inflammatory responses. Control animals received the same volume of endotoxin-free phosphate-buffered saline (PBS; vehicle). Relative qRT-PCR analysis demonstrated that the level of TNF-α mRNA was significantly up-regulated in the taste epithelium of mice injected with LPS compared with that in control animals. As shown in Figure 4F, the TNF-α mRNA level in LPS-treated mice increased to about 40-fold in taste epithelium (*P*<0.001) but did not significantly increase in nontaste epithelium (*P* = 0.29), compared with the levels in PBS-treated control mice. Consistent with these RT-PCR data, immunohistochemical analysis showed that the percentage of TNF-α-producing cells increased in taste buds of mice treated with LPS. As shown in Figure 4A–E, the number of TNF-α-immunoreactive cells in foliate and circumvallate taste buds was significantly increased (*P*<0.05 and *P*<0.01, respectively) in LPS-treated mice compared with PBS-treated control mice. These results indicate that the pro-inflammatory agent LPS further induced TNF-α expression and production when administrated systemically.

### LPS Activates TNF-α Production and Secretion by Taste Cells in vitro

**Figure 5:**
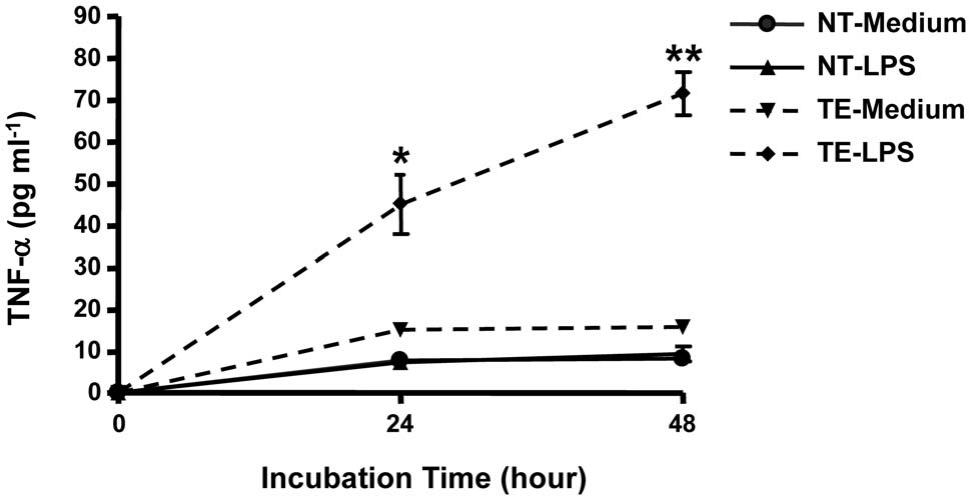
Production and secretion of TNF-α by taste tissue upon LPS challenge *in vitro*. Mouse taste epithelium (TE; circumvallate and foliate) and nontaste lingual epithelium (NT) were isolated and incubated in DMEM culture medium. The samples were treated with 5 µg ml^−1^ LPS for the time periods indicated. The cultures having no LPS in the medium served as controls. Concentrations of TNF-α in the supernatant of the cultured tissues were measured using ELISA. For each repeat of the experiment, tissues from three mice were used for medium control or LPS treatment. Each collected supernatant was assayed in duplicate for TNF-α concentration. The experiment was repeated three times, and the results were analyzed together. Data are mean ±SD. *P<0.01 (t = 4.3, df = 4), **P<0.001 (t = 9.8, df = 4), compared with medium control.

To investigate whether taste cells can produce and secrete TNF-α into their environment, we carried out an experiment to analyze TNF-α secretion from cultured taste tissues. Isolated mouse foliate and circumvallate epithelium, as well as the tongue epithelium devoid of taste buds, was cultured in medium with or without 5 µg ml^−1^ LPS for 24–48 h [23]. The amount of TNF-α in culture supernatants collected at different time points was measured by enzyme-linked immunosorbent assay (ELISA). As shown in Figure 5, the supernatant of cultured taste epithelium treated with LPS had significantly higher TNF-α level over the observed time period than did that of cultured taste epithelium without LPS treatment or cultured nontaste epithelium with or without LPS treatment (*P<0.01*). The results also showed a low but sustained level of TNF-α in the supernatant of taste epithelium without LPS treatment, which may reflect a basal level of spontaneous TNF-α secretion in taste buds. TNF-α levels in the supernatants of nontaste epithelium were barely detectable with or without LPS treatment. The data clearly demonstrate that taste cells can produce and secrete TNF-α in response to the inflammatory activator LPS.

### LPS-induced TNF-α Expression in the Taste Epithelium is TLR Dependent

**Figure 6:**
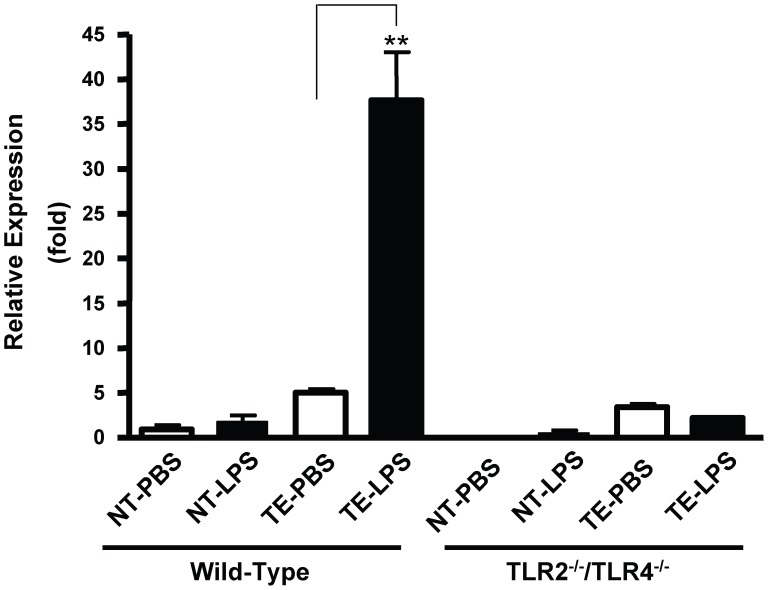
Absence of functional *TLR2* and *TLR4* genes attenuates LPS-induced TNF-α expression in taste buds. LPS (5 mg kg^−1^ body weight) was given to *TLR2^−/−^/TLR4^−/−^* double-knockout mice and wild-type C57BL/6 mice via i.p. injection. The same volume of endotoxin-free PBS (vehicle) was administrated to control animals. At 3 h post-LPS challenge, *TNF-α* mRNA levels in circumvallate- and foliate-containing taste epithelium (TE) and nontaste lingual epithelium (NT) were determined using qRT-PCR. Relative levels (fold) of *TNF-α* mRNA expression are shown. The expression level of *TNF-α* mRNA in nontaste tissue of wild-type mice receiving PBS was arbitrarily defined as 1. β-Actin served as the endogenous control gene for relative quantification. The same sets of 18 C57BL/6 mice described in Figure 4F were used as wild-type control mice. Parallel samples from *TLR2^−/−^/TLR4^−/−^* mice were prepared. Totally 18 *TLR2^−/−^/TLR4^−/−^* mice were used in this study. Data are mean ±SD. **P<0.001 (ANOVA with *post hoc* Dunnett tests, Mean squared error  = 21.6, df = 16).

We previously demonstrated the expression of several TLRs in taste bud cells, of which TLRs 2, 3, and 4 are preferentially expressed in a subset of taste cells [11]. TLR2 and TLR4 are cell surface receptors that recognize a variety of microbial-derived molecules and play essential roles in the initiation of inflammation. When the microbial components bind to TLRs, they trigger intracellular signaling pathways and induce the expression of a large number of cytokines, including TNF-α. To investigate the mechanism of TNF-α production in taste buds, we analyzed the role of TLRs in LPS-induced TNF-α expression. Although gram-negative-bacteria-derived LPS elicits immune responses primarily through TLR4, LPS preparations often contain contaminates that are TLR2 ligands [24]. To eliminate effects of possible contaminates in LPS, we performed the experiment using *TLR2^−/−^/TLR4^−/−^* double-knockout mice. LPS (5 mg kg^−1^ body weight) was administrated to *TLR2^−/−^/TLR4^−/−^* mice and wild-type C57BL/6 mice by i.p. injection. The levels of *TNF-α* mRNA in taste tissues at 3 h after LPS injection were assessed using qRT-PCR. As shown in Figure 6, LPS challenge significantly increased *TNF-α* mRNA level in the taste epithelium of wild-type animals but did not increase its level in *TLR2^−/−^/TLR4^−/−^* mice (ANOVA with *post hoc* Dunnett tests). In addition, there was a slightly increased level of *TNF-α* mRNA in nontaste tissue of wild-type mice treated with LPS compared with mice treated with PBS, but the increase was not statistically significant (*P*>0.05). In *TLR2^−/−^/TLR4^−/−^* mice, there was no detectable *TNF-α* mRNA in nontaste tissue of PBS-treated mice and a low but detectable level of *TNF-α* mRNA in LPS-treated mice. The results confirmed that the induction of TNF-α in taste buds by LPS is mediated through TLR-dependent pathways.

## Discussion

The current study demonstrates high levels of expression of TNF-α mRNA and protein in taste buds and identified the cell type that produces and releases TNF-α in taste buds. The results are important for further understanding the interactions between the peripheral taste system and immune activities, as well as the roles of taste cells in local immune responses in various diseases.

Real-time RT-PCR showed that *TNF-α* expression levels were higher in lingual epithelial tissues containing taste buds than in those without. Additionally, cells within the taste bud were immunoreactive to antibodies against TNF-α, whereas TNF-α-producing cells were not observed in nontaste lingual epithelium (Figure 1). These data demonstrate that taste buds are TNF-α-producing organs and that a high basal level of TNF-α is constitutively produced in these organs in untreated control mice. While we previously demonstrated production of TNF-α in circumvallate and foliate taste buds in an LPS-induced acute inflammation model [5], this is the first study documenting the presence of TNF-α in taste buds under physiological conditions. The presence of TNF-α in taste buds of control mice suggests a physiological or nonpathological function of this cytokine in taste tissues.

TNF-α activates various signaling pathways in immune cells, neurons, epithelial cells, and adipocytes [14]–[16]. TNF-α signaling can have destructive or protective effects on target cells depending on the expression of its receptors and downstream signaling components [25]–[28]. It is well known that taste cells undergo continuous turnover, during which aged taste cells degenerate and are replaced by new cells differentiated from basal cells [20], [29]. Our recent study suggested that LPS-induced inflammation could affect this process by inhibiting taste progenitor proliferation, interfering with taste cell renewal, and reducing the life span of taste cells in taste buds [5]. However, the underlying mechanisms remain unknown. Given that taste buds apparently contain a constitutive level of TNF-α, it may be possible for TNF-α to act as a general regulator of cell turnover by influencing cell death and survival.

There are four morphologically and functionally distinct types of taste cells in taste buds [20]. Type I cells comprise about half of the taste bud cells and have a glia-like function as they surround and support other cell types. Type II cells express several proteins involved in the transduction of sweet, umami, and bitter taste responses, including the G-protein gustducin and PLC-β2 [30], [31]. G-protein-coupled taste receptors T1Rs and T2Rs are expressed in nonoverlapping subsets of type II cells [32], [33]. Type III cells form conventional synapses with afferent gustatory nerves and express NCAM, and these cells seem to be important for sour and salty taste transduction [34]. A small number of type IV cells or basal cells are generally considered as immature taste cells that differentiate into other types of cells [20]. So far, our knowledge about the cellular origin of cytokines, including TNF-α, in the oral mucosa is still very limited, and information on the production of cytokines in taste tissue is almost completely lacking. Regarding TNF-α, we observed that only a subset of type II taste cells labeled by PLC-β2 produces TNF-α, while type I and type III cells do not produce TNF-α under both normal and inflammatory conditions (Figures 2, 4). The results show that a subset of type II taste cells exclusively produces TNF-α in taste buds and therefore may regulate immune responses in taste buds and in oral mucosa.

Interestingly, TNF-α in taste buds is co-expressed with T1R3 in a subset of type II taste cells. In circumvallate and foliate taste buds, TNF-α is co-expressed with T1R3 but not with gustducin (Figure 3). Thus, PLC-β2-expressing type II taste cells are heterogeneous in terms of TNF-α production, and at least two types of type II taste cells exist in circumvallate and foliate papillae: PLC-β2^+^T1R3^+^TNF-α^+^ and PLC-β2^+^gustducin^+^TNF-α^–^. The results indicate that a specific subset of type II cells that exclusively produce TNF-α in taste buds is closely associated with T1R3 expression. Recent studies have shown that T1R3 can form a functional heterodimeric receptor with T1R1 or T1R2 to detect umami or sweet substances [35], [36]. T1R2/T1R3 heterodimers can be activated by sweet-tasting compounds at physiological concentrations. T1R1/T1R3 heterodimers can be activated by L-amino acids such as monosodium glutamate (MSG). Co-expression of TNF-α with T1R3 raises the possibility that TNF-α and taste signaling pathways, especially those for sweet and umami, may mutually regulate or interact with each other within taste buds.

Can TNF-α-expressing taste cells respond to microbial-derived molecules, such as LPS? This question is important for understanding the roles of TNF-α produced by taste cells in pathological processes under noxious challenges including infection, injury, chemo- and radiotherapy, and stress. The results of this study show that TNF-α and its mRNA are significantly up-regulated in taste tissue of mice receiving systemic LPS administration (Figure 4). This finding indicates that the TNF-α-producing cells in taste tissues indeed can respond to inflammatory stimuli, similarly to the TNF-α-producing immune cells such as macrophages. However, the production of TNF-α in taste cells does not necessarily indicate that the produced TNF-α would be displayed on the cell surface or secreted into the local environment, which is necessary for TNF-α to act on target cells. To examine the secretion of TNF-α from taste tissue, we designed experiments to challenge the tissue in vitro with LPS in a tissue cultural system. The data verified the ability of taste cells to secrete TNF-α in response to inflammatory activators (Figure 5). These results suggest that TNF-α produced by taste cells may function as a physiologic or pathological mediator in immune surveillance and inflammation.

Expression and secretion of TNF-α in response to LPS require a series of signaling events, and in recent years these details have been extensively studied in macrophages and microglial cells [37], [38]. The initiation of this pathway requires the binding of LPS to its cognate transmembrane receptors CD14 and TLR4, which leads to the activation of NF-κB [39]. In this study, in order to understand the mechanism of the LPS-induced TNF-α production and secretion in taste buds, we analyzed changes in *TNF-α* mRNA levels in *TLR2^−/−^/TLR4^−/−^* double-knockout mice following LPS injection. Although TLR4 is the primary receptor that mediates the signaling pathways of LPS from gram-negative bacteria, we used *TLR2^−/−^/TLR4^−/−^* double-knockout mice in this study to eliminate the possible effects from TLR2 ligands that may be present as contaminates in LPS preparations [24]. The results confirmed that the induction of TNF-α production in taste buds initialized by LPS is mediated through TLR pathways, because absence of TLR2 and TLR4 eliminated LPS-induced TNF-α expression in taste tissues (Figure 6).

In summary, we show here that a specific subset of taste cells selectively expresses high levels of TNF-α in apparently healthy adult mice. These cells can rapidly increase TNF-α production upon LPS challenge, both *in vivo* and *in vitro,* via TLR-mediated pathways. These results will help us to understand the impact of inflammation on taste perception and the interactions between the immune and gustatory systems.

## Materials and Methods

### Ethics Statement

This study was performed in strict accordance with the recommendations in the Guide for the Care and Use of Laboratory Animals of the National Institutes of Health. All experiments involving animals were performed according to the protocol approved by the Monell Chemical Senses Center Institutional Animal Care and Use Committee (approved protocol # no.1122).

### Animals

Six- to eight-week-old adult male or female C57BL/6 (B6) mice were obtained from Jackson Laboratory (Bar Harbor, ME). *TLR2^−/−^* and *TLR4^−/−^* mice were generated at the Department of Host Defense, Osaka University, Osaka, Japan [40], [41]. *TLR2^−/−^/TLR4^−/−^* double-knockout mice were generated by crossing *TLR2^−/−^* and *TLR4^−/−^* mice [42]. These mice have been backcrossed to C57BL/6 background. T1R3-GFP mice, expressing GFP under the control of a mouse T1R3 promoter, were generously provided by Dr. R. Margolskee’s laboratory [43]. Mice were housed and maintained in strict accordance with the guidelines for the management of laboratory animals at the Monell Chemical Senses Center. The animals were housed three to four per cage, maintained in a room under a 12/12 h light/dark cycle, and given free access to standard rodent food (8604 Teklad rodent diet, Harlan Laboratories) and water. Mice were housed in a regular facility (not a pathogen-free facility). Control mice used in all experiments appeared healthy with no visible symptoms of infection. All procedures were done in accordance with protocols approved by the Institutional Animal Care and Use Committee of Monell Chemical Senses Center.

### Reagents

A goat polyclonal antibody against mouse TNF-α was purchased from R&D Systems. (Minneapolis, MN). A rabbit polyclonal antibody against mouse ectonucleotidase nucleoside triphosphate diphosphohydrolase 2 (ENTPDase2) was purchased from Centre de Recherche (Quebec, Canada). Rabbit polyclonal antibodies against PLC-β2 (sc-206) and gustducin (sc-395) were purchased from Santa Cruz Biotechnology (Santa Cruz, CA). A rabbit polyclonal antibody against neural cell adhesion molecule (NCAM) was purchased from Chemicon (Temecula, CA). A purified rabbit antibody against mouse T1R3 was kindly given by Drs. Peihua Jiang and Robert Margolskee. Dylight-649-conjugated donkey anti-rabbit or anti-goat antibody and Dylight-488-labeled donkey anti-goat antibody were purchased from Jackson ImmunoResearch Laboratories (West Grove, PA). Rat antibodies against mouse CD3 and CD11b were purchased from BD Pharmingen (San Diego, CA). Collagenase A and dispase II were from Roche Applied Sciences (Indianapolis, IN). LPS (from *Escherichia coli* O111:B4) was purchased from Sigma (St. Louis, MO).

### Immunohistochemistry

Tongue tissue processing and immunostaining were carried out as previously described [4], [5]. Briefly, excised mouse tongue tissues were fixed in freshly prepared 4% paraformaldehyde in PBS for 1 h on ice and then cryoprotected in 20% sucrose/PBS solution at 4°C overnight. Tissues were sliced into 10-µm-thick sections using a Microm HM 500 OM cryostat (Thermo Scientific Microm, Walldorf, Germany). Circumvallate and foliate sections were cut in parallel to the surface of the tongue. Fungiform sections were collected from the tip of the tongue, which was cut coronally. For immunostaining of TNF-α, fixed tissue sections were washed 3x with PBS containing 0.3% Triton X-100 and then incubated with a permeabilization buffer containing 0.1% saponin and 0.009% sodium azide (eBioscience, San Diego, CA) at room temperature for 1 h, followed by incubation with blocking buffer (3% bovine serum albumin, 0.3% Triton X-100, 2% horse serum, and 0.1% sodium azide in PBS) containing 0.1% saponin at room temperature for 1 h. The sections were then incubated with affinity-purified goat antibody against TNF-α in the blocking buffer (1∶200) at 4°C overnight and then incubated with Dylight-488-conjugated donkey anti-goat antibody at room temperature for 1 h. For colocalization studies, the TNF-α antibody together with rabbit antibody against PLC-β2 (1∶500), NCAM (1∶200), ENTPDase2 (1∶500), gustducin (1∶1000), or T1R3 (1∶500) was applied as primary antibodies following the same procedure as TNF-α-only immunostaining. The secondary antibodies Dylight-488-conjugated donkey anti-goat antibody and Dylight-649-conjugated donkey anti-rabbit antibody were used for these experiments. Images were taken using a Leica confocal microscope. For control experiments, we replaced TNF-α antibody with nonspecific normal goat IgG, followed by incubations with the same secondary antibody mentioned above. For antigen blocking experiments, TNF-α antibody was preincubated with purified recombinant TNF-α (PeproTech, Rocky Hill, NJ). These mixtures were then added to the tissue sections and processed as described above.

For immunohistochemistry using anti-CD3 and anti-CD11b antibodies, we followed procedures described previously [9]. Briefly, frozen sections were dried at room temperature for 30 min and rehydrated for 10 min in 0.1 M PBS at pH 7.0. Endogenous peroxidase activities were blocked by 3% hydrogen peroxide for 10 min and then washed 3x with PBS. The sections were then incubated in Superblock (Thermo Scientific, Rockford, IL) for 1 h at room temperature. Antibodies against CD3 and CD11b were incubated with the tissue sections for 2 h at room temperature. After three 5-min washes, tissue sections were incubated for 1 h with a secondary biotinylated antibody and then with prediluted streptavidin–horseradish peroxidase (HRP) for 45 min. Immunoreactivity was detected using diaminobenzidine (DAB) as the chromogen. Controls for nonspecific binding were performed by excluding primary antibodies. Brightfield images were captured using a Nikon DXM-1200C attached to a Nikon Eclipse 80i microscope.

### Cell Counting

To quantify the number of TNF-producing cells in taste tissue, two circumvallate and foliate sections were selected for immunostaining from serial sections of each papilla. These sections were chosen from the middle range of papillae, but separated from each other by at least 40 µm (four 10-µm-thick sections away). The numbers of TNF-producing cells and PLC-β2-positive cells were counted from confocal images of double immunostaining as described above. PLC-β2-positive cells and TNF-producing cells were counted from single-channel images, and TNF- and PLC-β2-double-positive cells were counted from overlay (or merged) images. Only cells with staining clearly above the background and with taste cell morphology (spindle-shaped) and/or with obvious nuclei were counted as positive cells. Double-labeled cells were discriminated from cells that may simply be overlapping one another by morphology and by judging from serial images taken along the z-axis of individual tissue sections (serial scanning). The percentage of TNF-producing cells among PLC-β2-positive cells was then calculated for each mouse. Five mice were included in each group of the experiments.

### Isolation of Mouse Taste Epithelium

Freshly collected tongues were rinsed 3x in Ca^2+^-free Tyrode’s solution (140 mM NaCl, 5 mM KCl, 10 mM HEPES, 10 mM glucose, and 10 mM Na-pyruvate) containing 10x Antibiotic-Antimycotic (Life Technologies, Grand Island, NY). After transfer to a new dish, tongues were injected with an enzyme solution (2 mg ml^−1^ dispase II, 1 mg ml^−1^ collagenase A in Ca^2+^-free Tyrode’s solution) just beneath the epithelium and incubated at 37°C for 15–20 min. Whole epithelium was then peeled off from the posterior end of the tongue using two pairs of forceps and rinsed 3x in Tyrode’s solution containing 1x Antibiotic-Antimycotic. The circumvallate and foliate epithelia containing taste buds were separated carefully from the nongustatory epithelia and placed in cultural medium for tissue culture, or in lysis buffer for RNA preparation and qRT-PCR analysis. The whole process was performed under a dissecting microscope.

### qRT-PCR

Mice were euthanized and the tongue epithelium was prepared as previously described [4]. Total RNA was extracted using Absolutely RNA Microprep Kit (Stratagene, Cedar Creek, TX). Epithelial pieces containing foliate or circumvallate taste buds from three mice in each group were pooled as the taste epithelium sample. Approximately equal amounts of total RNA from these samples were reverse transcribed into cDNA using Superscript III reverse transcriptase (Technologies, Grand Island, NY). qPCR was set up using Power SYBR Green PCR Master Mix (Applied Biosystems, Foster City, CA) and analyzed on an ABI PRISM 7000 Sequence Detection System (Applied Biosystems). Relative quantification of gene expression was performed using ABI software, which was based on the 2^−ΔΔCt^ method [4]. β-Actin was used as the endogenous control gene for these analyses. The experiments were repeated three times. RT-PCR primers for TNF-α were: forward CCACATCTCCCTCCAGAAAAGA and reverse GCTGGGTAGAGAATGGATGAAC. Primers for β-actin were forward GATTACTGCTCTGGCTCCTA and reverse ATCGTACTCCTGCTTGCTGA.

### Cultures of Taste Tissues

Isolated foliate and circumvallate taste buds from three mice in each group were pooled as the taste epithelium sample. The same amount of peeled-off nontaste epithelial pieces from the same animals (excised from the back of the tongues) was also collected as nontaste tissue control. To minimize microbial contamination, the collected tissues were incubated in 5x Antibiotic-Antimycotic (GIBCO)-containing culture medium for 20 min on ice. Following 3x washes in Dulbecco’s modified Eagle’s medium (DMEM), the tissues were cultured at 37°C in DMEM containing 10% fetal bovine serum and 1x Antibiotic-Antimycotic in a of 5% CO_2_ atmosphere. LPS (5 µg ml^−1^) was added to the treatment group [23]. The supernatants were collected at designated time points and were stored at −80°C before TNF-α ELISA.

### ELISA for TNF-α

Levels of TNF-α in culture supernatants were measured by ELISA, using Mouse TNF-α ELISA Ready-SET-Go! Kit® (eBioscience), according to the manufacturer’s recommendations. Briefly, the 96-well ELISA plates were coated at 4°C overnight with 100 µl capture monoclonal antibody per well in 0.2 M sodium phosphate buffer (pH 6.5) and then blocked with 200 µl assay diluent at room temperature for 1 h. Samples were loaded in duplicates in 100 µl assay diluent, and the plates were incubated at room temperature for 2 h. Serial two-fold dilutions of recombinant TNF-α were carried out and used as standards. A 100 µl volume of biotin-conjugated detection antibody was added to each well and incubated for 1 h at room temperature. After washing, 100 µl of avidin-HRP was added to each well. Finally, a mixture of tetramethylbenzidine and hydrogen peroxide was added as a substrate for HRP, and the color in wells was allowed to develop at room temperature for 30 min in the dark. The reaction was stopped by addition of 2 N H_2_SO_4_. The optical density value at 450 nm was measured using Flex Station 2. TNF-α concentrations in different groups were determined by comparison with the standard curve.

### Statistical Analysis

Most statistical analysis was done by two-tailed unpaired Student’s *t* tests using the GraphPad PRISM computer program (GraphPad Software, La Jolla, CA). Statistical analysis of TNF mRNA levels in wild-type and *TLR2^−/−^/TLR4^−/−^* mice was done by factorial ANOVA using Statistica software (StatSoft, Inc., Tulsa, OK). *Post hoc* Dunnett and Fisher LSD tests were then performed to compare TNF mRNA levels between different tissues (taste vs. non-taste), treatments (PBS vs. LPS), and mouse strains (wild-type vs. *TLR2^−/−^/TLR4^−/−^* knockout mice). *P*-values <0.05 were considered statistically significant.

## Supporting Information

Figure S1 **Colocalization patterns of T1R3 and gustducin in circumvallate, foliate, and fungiform taste papillae.** T1R3 and gustducin are co-expressed in fungiform but are mostly segregated in foliate and circumvallate taste buds. Confocal images of immunofluorescent staining of gustducin on tissue sections from circumvallate (CV), foliate, and fungiform (FF) papillae of T1R3-GFP transgenic mice. T1R3-GFP-positive cells are shown in green and gustducin-positive cells are shown in red. Scale bars: 35 µm.
(TIF)
