# Supplementary figures and images for: Expression and Secretion of TNF-α in Mouse Taste Buds: A Novel Function of a Specific Subset of Type II Taste Cells
Source: PLoS One. 2012 Aug 14;7(8):e43140. doi: 10.1371/journal.pone.0043140 (PMC3419207; doi:10.1371/journal.pone.0043140)

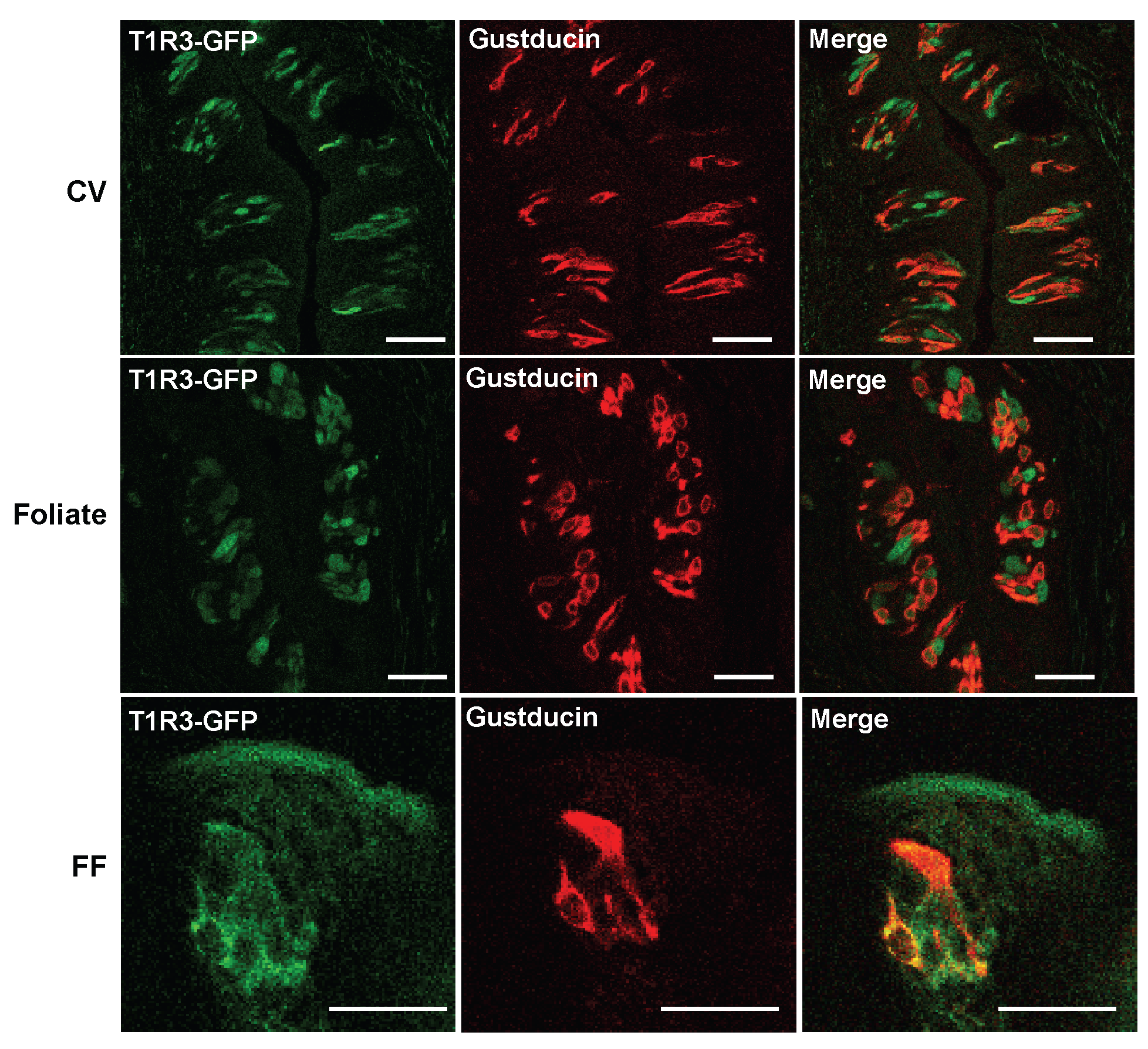

Supplement: Figure S1 — Colocalization patterns of T1R3 and gustducin in circumvallate, foliate, and fungiform taste papillae. T1R3 and gustducin are co-expressed in fungiform but are mostly segregated in foliate and circumvallate taste buds. Confocal images of immunofluorescent staining of gustducin on tissue sections from circumvallate (CV), foliate, and fungiform (FF) papillae of T1R3-GFP transgenic mice. T1R3-GFP-positive cells are shown in green and gustducin-positive cells are shown in red. Scale bars: 35 µm. (TIF) [file pone.0043140.s001.tif]
